# Multiscale anthropogenic feature detection in the Argentinian Andes: satellite machine learning, UAV, and pedestrian survey

**DOI:** 10.1038/s40494-026-02813-z

**Published:** 2026-07-13

**Authors:** Manuel J. H. Peters, Amina Jambajantsan, Veronica Zuccarelli Freire, Laura Pey, José María Vaquer, Patrick Roberts

**Affiliations:** 1 Department of Coevolution of Land Use and Urbanisation, Max Planck Institute of Geoanthropology, Jena, Germany; 2 School of Archaeology, University of the Philippines, Quezon City, Philippines; 3 Department of Geography, Ghent University, Ghent, Belgium; 4 MAPSS, Max Planck Institute of Geoanthropology, Jena, Germany; 5 Consejo Nacional de Investigaciones Científicas y Técnicas - Instituto Regional de Estudios Socio-culturales (IRES-CONICET/UNCA), Universidad Nacional de Catamarca, Catamarca, Argentina; 6 Consejo Nacional de Investigaciones Científicas y Técnicas (CONICET) - Instituto de Arqueología, Facultad de Filosofía y Letras, Universidad de Buenos Aires, Buenos Aires, Argentina; 7 Institut für Orientalistik, Indogermanistik, Ur- und Frühgeschichtliche Archäologie, Friedrich Schiller Universität, Jena, Germany

## Abstract

The Cusi Cusi micro region in Argentina’s Andes presents a unique challenge for cultural heritage research. This rugged area lies within the Puna of Jujuy at elevations from 3800 to 4200 metres above sea level. Archaeological, historical, and features indicate long term human presence, including livestock enclosures, agricultural terraces, and settlement structures. The region is situated within the ‘Lithium Triangle’ and faces challenges in resource ownership, Indigenous rights, and landscape change, highlighting the importance of documenting cultural and environmental heritage. We present an integration of satellite and unpiloted aerial vehicle (UAV) imagery with pedestrian survey datasets, machine learning (ML), and physical ground truthing to explore anthropogenic activities in the landscape. Satellite-based ML detects features while UAV data improves resolution and assessment of present activities. Targeted pedestrian survey validates results and provides potential dating. This combined approach identifies loci of activities and offers a framework for investigating other rugged regions.

## Introduction

There is a growing interest in documenting the nature and extent of past human presence and land use across the globe, especially given its relevance for cultural heritage and long-term human-environment relationships^1,2^. South America has been particularly pivotal in these discussions in recent years, with emerging evidence for long-term human occupation of, and interaction with, what have often been considered ‘pristine’ environments^3^. For example, there has been growing recognition of the scale of human presence within the Amazon rainforest, ranging from early examples of cultivation^4^ to the formation of Indigenous ‘Garden Cities’^5,6^, with corresponding impacts for understanding the long-term role of humans in shaping contemporary ecosystems and landscapes. Remote sensing approaches have played a particularly important role in re-evaluations of human presence in tropical forest contexts which have been traditionally challenging for survey as a result of their remoteness, difficult working conditions, or barriers to visibility^6^.

**Fig. 1:**
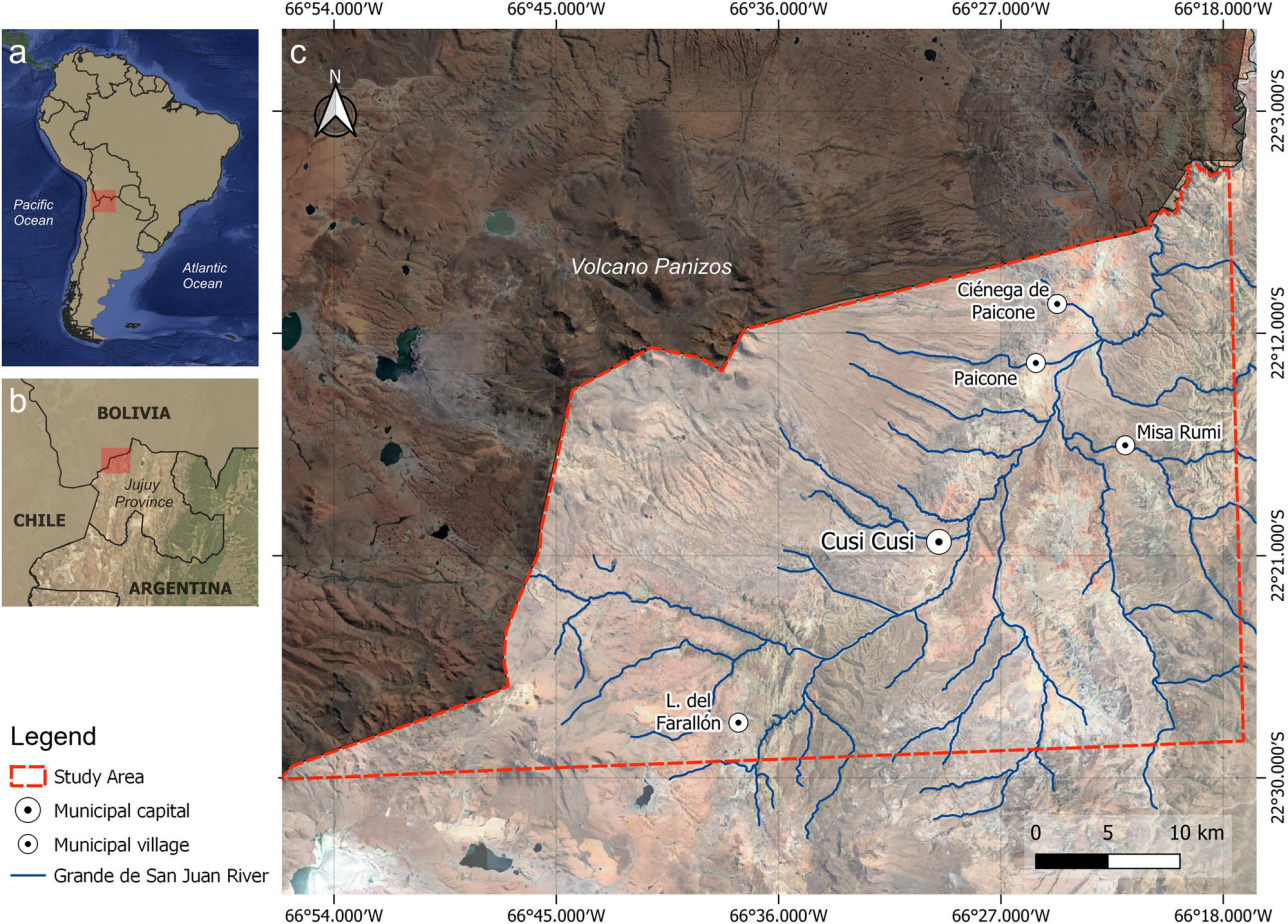
Location of the Cusi Cusi region and the study area. The study area is shown within South America. (**a**), within Argentina (**b**), and within the Cusi Cusi study area (**c**). The red dashed line marks the study area, the large circle the municipal capital, the smaller circles municipal villages, and the blue lines the Grande de San Juan River. Background imagery: Maxar.

These approaches also have the potential to enrich our understanding of human-landscape interactions in other ‘extreme’ contexts such as deserts, high-altitude areas, and steppe conditions^7,8^, threfore these contexts are increasingly being explored from a remote sensing perspective in South America^9^. In the rugged terrain of the Argentinean Andes, the Cusi Cusi micro-region (Fig. 1) presents a unique challenge for archaeological research. Located within the Puna of Jujuy, at an elevation ranging from approximately 3800 to 4200 metres above sea level (masl), this area is marked by plateaus interrupted by sharp elevation changes. This high-altitude area contains long-term human occupation traces including archaeological, historical, and modern features, such as habitational structures, burial structures, agricultural terraces, retaining walls, perimeter walls, and enclosures for herding practices^10^. In this study, we focus on documenting dispersed enclosure features such as corrals across the landscape.

Cusi Cusi has yielded archaeological evidence of human presence dating back to the Middle Holocene (7000–5000 years BP, relatives dates on diagnostic lithic materials), associated with the transition from a hunter-gatherer lifestyle to a pastoral way of life. Around the 13th century AD, populations from the Doncellas-Casabindo region settled in the area, creating an agricultural landscape on a scale not seen before in this region^10^. This continued until the 16th century AD with the Spanish invasion, when agricultural sites were abandoned, and populations returned to pastoralism as their main means of subsistence^11–13^. Each of these lifestyles, along with their associated land use strategies, reflects changes and continuities in the relationships between human populations and the environment^10,14^. To explore and document these dynamics in detail, it is essential to develop survey methodologies that record material culture and spatial relationships associated with each way of life across broad spatial and temporal scales.

The context of the Cusi Cusi area is further complicated by the 2023–24 provincial constitution reforms and the new national regimen for foreign investments (known as RIGI), which have significantly altered the dynamics concerning ownership of underground resource ownership and Indigenous rights over their ancestral land. The Puna of Jujuy is situated in the ‘Lithium Triangle’; the wider region within Argentina, Bolivia, and Chile that holds 58% of the lithium in the world^15^. The presence of this resource and other minerals has made it a focal point for extractive industries. This increasing economic interest and the connected politics have added another layer of intricacy to the already complicated situation regarding Indigenous land ownership, particularly in contexts where communities hold *de facto* or privately recognised tenure but lack formal land titles, leaving them legally vulnerable to external claims and development pressures^16^. In addition, the potential consequences of large-scale lithium mining are posing a significant threat to the environmental stability of the region as well as the preservation of its cultural heritage. This underscores the increasing importance of documenting heritage in the region, particularly concerning long-term records of land use and their connection to increasingly threatened Indigenous ways of life.

Pedestrian survey, combined with community interaction, arguably provides the highest-resolution data, but it significantly limits the spatial range of investigation, particularly in challenging terrain at high altitudes^10^. Additionally, there may be features that are not commonly recognised on foot, such as subtle irrigation channels^17^. In such cases, alternative data sources such as satellite data and aerial imagery can provide an alternative perspective^18,19^. Although the application of satellite data now allows the study of unprecedentedly large areas, the available resolution of the data is often not sufficient for in-depth assessment and identification of individual features^20,21^. High-resolution historical aerial images collected by airplane can be used to supplement this data while providing wider views of a research area and contributing to deeper analysis of landscape change^22^. However, the coverage of these historical flights is limited in remote areas. In these regions, various types of validation data collected by UAV, e.g., videos, photographs, and orthophotography, provide an important addition. In areas where features are suspected to be present, or in research areas of limited size, UAV flights can be used to provide a higher resolution view. Unfortunately, UAVs generally have a rather limited flight time and require a targeted deployment, which assumes some prior knowledge of the location of features or areas of interest.

Here, we present the results of combining survey methods that allowed us to expand the record of cultural heritage sites in the region. Over the years, we have built a database of sites surveyed during our explorations in the area. These surveys were carried out in collaboration with the Indigenous Community ‘Orqho Runas’^10^. Other methods involved the analysis of satellite images and the distribution of archaeological sites in the landscape.

**Fig. 2:**
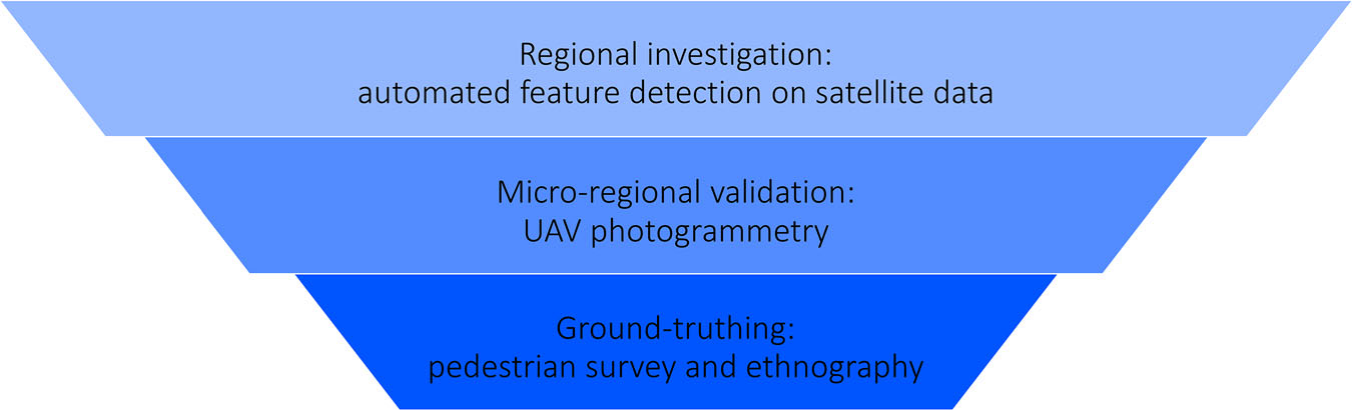
Multiscale workflow employed in this study. The figure summarises the workflow used to integrate regional investigation, micro-regional validation, and ground-truthing by combining automated feature detection on satellite data, unpiloted aerial vehicle (UAV)-based photogrammetry, pedestrian survey, and ethnography.

To address the potentials and limitations of these different approaches, we employ a multiscale approach, integrating satellite and UAV imagery with ML algorithms for automated feature detection (Fig. 2). Based on Indigenous knowledge, previous pedestrian survey data, and satellite aerial imagery, we determined an area of interest for the UAV validation data that included known sites as well as less-explored regions. Validation of the satellite remote sensing data was carried out by a combination of UAV-based orthophotography and on-site ground-truthing fieldwork. This integration of techniques allows us to gain additional insights into the region’s past and present. This comprehensive approach not only shed light on the archaeological heritage of the Cusi Cusi micro-region but also provided information regarding the contemporary human-environment interaction of the Indigenous communities.

The study area is situated in the upper basin of the Río Grande de San Juan, a river that flows through the Altiplano region along the border between northwestern Argentina and southwestern Bolivia. The environment corresponds to the Puna Seca, a high-altitude semi-desert traversed by mountain ranges that are oriented northeast to southwest. The altitude of the area varies between 3600 and 5700 masl. The climate of the area is cold and dry, with average annual temperatures of 3–6 °C, high evapotranspiration, high solar radiation and a wide daily temperature ranging from 30 °C during the day to night temperatures below −20 °C in the coldest months. It is a highly fragmented environment where the only available water sources are the few permanent freshwater catchments and small springs scattered across the landscape. Rainfall is highly seasonal, with at least 80% of rain falling between December and March, ranging from 200 to 400 mm per year^23,24^.

Rainfall in the region varies along both altitudinal and latitudinal gradients, and, combined with altitude-driven thermal differences, creates significant environmental heterogeneity. The result is a mosaic landscape of discontinuous vegetation patches. The area is part of the Puna phytogeographic province, where xerophytic vegetation is well adapted to the lack of water, low temperatures, and grazing pressure^25^. This limited vegetation cover generally affords high visibility of the surface archaeological record, facilitating both pedestrian survey and remote sensing. In areas with more extensive vegetation cover, active (laser-based) remote sensing would be appropriate. For the purposes of this pilot study, however, in an area with limited vegetation cover, passive (photo-based) remote sensing proved sufficient. Geologically, the micro-region lies within the Panizos Volcanic Complex, which includes two main lithological groups: the Panizos Dacite Group, made up of dacitic lavas, and the Panizos Ignimbrite Group. The latter consists of simple cooling ignimbrites, dacitic composites, and crystal-rich deposits; up to 50% consists of pumice^26^.

Previous work carried out in the Cusi Cusi region has identified more than 220 archaeological sites with diverse chronologies^10^. To organise them, we classified them into various *landscape logics* corresponding to the prevailing ways of life during different periods of human history in the region^11,13,27^. The earliest, the hunter-gatherer logic, developed from the beginning of the Holocene to the Middle Holocene, and is characterised by lithic assemblages linked to the domestication of camelids. The environmental conditions of the Cusi Cusi microregion during the Middle Holocene were likely favourable for the interactions between human populations and camelids, which resulted in ‘mutual domestication’^28^. This period marks the gradual transition towards a pastoral logic, as herding camelids became a primary subsistence strategy, a practice that continues into the present.

In the 13th century AD, populations from the Doncellas-Casabindo region settled in the area, significantly changing the pastoral landscape. In the surrounding ravines, where permanent and seasonal water sources are found, they established an agricultural-ritual landscape designed for production beyond the household scale^12,29^. Agriculture relied primarily on dry farming techniques, including main and secondary canals and runoff control walls, while the irrigation systems at Huayatayoc Alto were fed by at least one reservoir located over a natural spring. Additionally, erosion control strategies in gullies have been recorded in the Pajchela and Casas Quemadas ravine. This agricultural–pastoral system continued throughout the 14th and 15th centuries, when the area became incorporated into the Inca Empire, and persisted until the Spanish invasion, before becoming disarticulated around the 16th AD. At that time, the pastoral populations appropriated the agricultural sites, modifying them for livestock use and domestic production. The imposition of the colonial mode of production was centered on mining exploitation, and forced pastoral populations into market economies. One of the most important activities carried out by Indigenous peoples was livestock herding, which served as a food source for mine workers^30,31^.

The interpretative model we present reveals the social processes and land use based on the sites we have studied. However, large portions of the territory remain undocumented, and several key questions about the occupation sequence still need to be addressed. Among these, we can highlight the present knowledge gaps: 1. the specific role of the microregion during the transition from hunting and gathering to pastoral life, as the area has been proposed as a potential centre of camelid domestication in South America; 2. the kind of relationships between agricultural and pastoral populations between the 13th and 16th centuries AD; 3. the nature of the Inka presence in the region; 4. the impacts of the Spanish invasion on the ways of life of the Indigenous peoples. In addition to these questions, there are urgent concerns related to the growing threat of mining extractivism and the role of cultural heritage in shaping the identities of local Indigenous communities, even though Indigenous land tenure processes are covered under Argentinian constitutional rights and territorial ancestral land rights (Argentine law No. 26.160). Developing and expanding new remote sensing-assisted survey approaches will allow us to document and highlight this heritage, which in turn can help counter extractive pressures and support local communities’ demands for material and symbolic resources. In this study, we focus on evidence related to pastoral-agricultural logic, primarily residential complexes with associated enclosures scattered across the landscape. This pastoral-agricultural evidence, which links past records with present-day practices, is the most widespread in the study region and serves as a pilot case for our methodological approach.

## Methods

### Combination of approaches

Based on extensive field experience in the study area, we were able to identify how the target enclosure features manifest in satellite imagery. This empirical knowledge formed the basis for the selection of training data through visual identification of enclosure features in the satellite imagery. The initial training set included features that had been previously documented through pedestrian surveys, primarily located in the central part of the research area (Fig. 2). To account for the broader range of geomorphological and land use variability present across the region, additional enclosure features were manually identified in satellite imagery covering a wider spatial extent. Together, these samples provided a representative training set capturing the variability of enclosure features expected in this landscape.

**Fig. 3:**
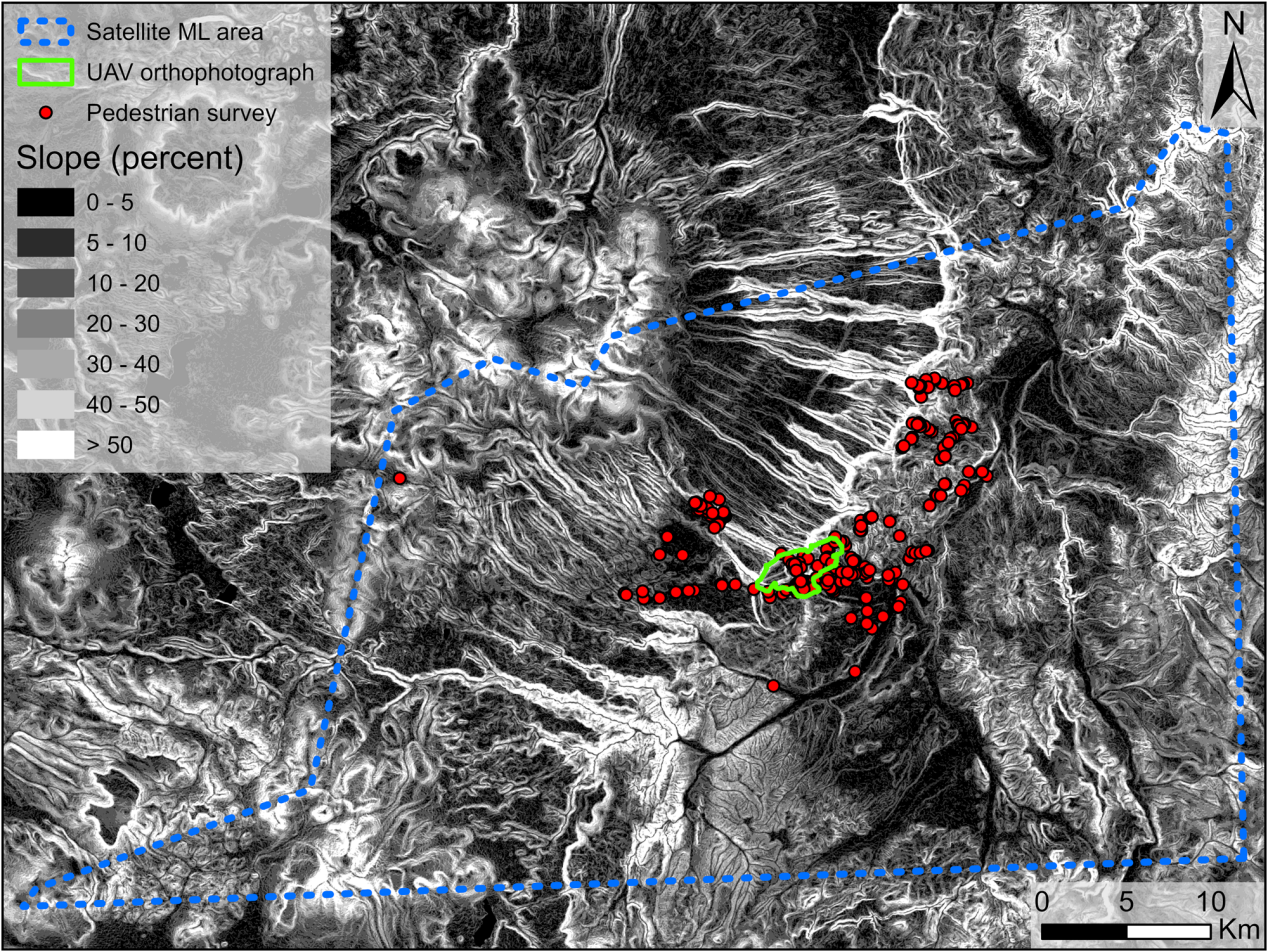
Slope percentage in the research area and coverage of the pedestrian survey, satellite, and unpiloted aerial vehicle (UAV) data. The figure shows slope percentage across the research area together with the spatial coverage of the datasets used in this study. The blue dashed line marks the area covered by satellite-based machine learning (ML), the green line marks the UAV-based orthophotograph, and the red dots show sites recorded during pedestrian surveys. The greyscale background represents slope percentage, ranging from 0 to 5% in black to more than 50% in white.

In addition to the ML, we carried out additional targeted pedestrian surveys in the central part of the region of interest, focusing on locations newly identified by the machine learning procedure. These surveys aimed to confirm the presence of archaeological features on the ground and to provide empirical validation for a subset of the detected features. Owing to the rugged terrain and limited accessibility of the study area, particularly the high elevation and the steep slopes (Fig. 3), comprehensive coverage through pedestrian survey alone is not feasible. This constraint constitutes a primary motivation for the integrated methodological approach adopted in this pilot study.

In several areas, the satellite imagery did not permit confident visual identification of features, despite their detection by the machine learning model. To address this limitation, high-resolution UAV imagery was acquired over a central region of interest (Fig. 3). These data provided a detailed and up-to-date visual reference, offering substantially higher spatial resolution than satellite imagery and an aerial perspective that helped bridge ground-level observations and broader landscape patterns. The UAV imagery also introduced an additional temporal reference for feature assessment. Taken together, the integration of pedestrian survey, UAV photogrammetry, and satellite-based machine learning enabled a complementary, multiscale approach to the documentation and interpretation of the cultural landscape.

### Satellite data collection and ML

**Fig. 4:**
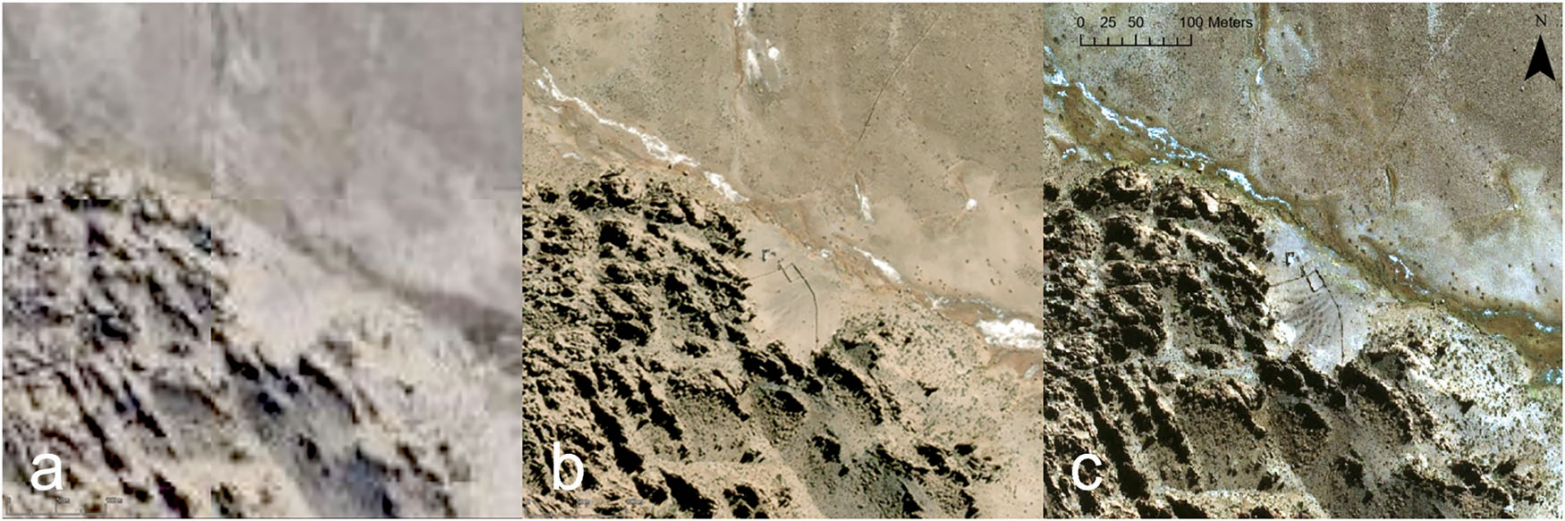
Google, Bing, and Maxar satellite images of the same region accessed through SAS Planet. The Google (**a**), Bing (**b**), and Maxar (**c**) images illustrate differences in resolution, contrast, surface visibility, and the detectability of archaeological and landscape features between the three satellite image sources.

Initial data were collected by means of satellite images provided by Google, Bing, and Maxar and obtained through the open access platform SAS Planet (https://github.com/sasgis/sas.planet.src). Based on project requirements, a region of interest of roughly 1750 square kilometres was selected (Fig. 1). Imagery resolution varied: Bing and Maxar provided scenes at around 60 cm resolution, whereas Google imagery lacked sufficient detail for object detection tasks (Fig. 4). Imagery availability in the high Andes is strongly biased toward the dry season, because the wet season is associated with increased cloud cover, which reduces the number of cloud-free scenes suitable for mapping. Conveniently, vegetation cover is also at a low during the dry season, which is advantageous for mapping purposes. Due to its superior resolution in this region (Fig. 4), Maxar imagery was selected for the ML procedure. Only visible light spectrum imagery (RGB) was used, since the resolution of multispectral data in this region was not sufficient for our purposes, and RGB contained all information required for this study.

We employed a deep learning approach, a subset of machine learning that enables the automated processing of unstructured spatial data for the detection and classification of archaeological features^32,33^. The ML component of this study focused specifically on identifying enclosure features, since these leave distinct and recognisable anthropogenic traces in the landscape, visible at the available resolution. Moreover, this type of evidence is closely associated with the agro-pastoral societies that this research seeks to characterise. These features formed the basis of the training dataset used in the detection algorithm. All spatial data were processed using the EPSG:4326 (WGS84) coordinate reference system. ML analyses and visualisation of results were carried out using ArcGIS Pro 2.9.11.

After acquiring the highest-resolution open-source imagery available (60 cm/pixel), we created training data with corresponding labels for enclosure features. These training data were based on previously identified and validated enclosures within the study area. Owing to the rugged character of the terrain, earlier fieldwork was necessarily restricted to more accessible parts of the landscape. GPS coordinates from these surveys were used to locate corresponding enclosure features in the satellite imagery. In total, 50 georeferenced features from these accessible areas were included in the training dataset. To broaden the range of landscape contexts represented, an additional 50 features were identified through visual inspection of satellite imagery in more remote areas, where pedestrian access was limited. The classification dataset comprises a single target class (“Enclosure”), defined based on morphologically enclosed structures, such as corrals, visible in satellite imagery. Non-enclosure landscape features (e.g., bedrock exposures, riverbeds, erosion features, and anthropogenic structures) are implicitly represented as background within the training image chips. This approach follows standard object-detection practice, where the model learns to discriminate target objects from heterogeneous background rather than from explicitly labelled negative classes.

A total of 100 training samples were manually annotated, and several data augmentation strategies were applied to increase robustness to variability in feature orientation, illumination, and scale. These included random rotations (±30°), brightness adjustments, contrast variations, and random zooming. Together, these augmentations simulate differences in acquisition geometry, lighting conditions, and spatial scale commonly encountered in high-resolution satellite imagery and help reduce overfitting. The generated images were formatted according to the PASCAL Visual Object Classes (VOC) and R-CNN Mask metadata standards, both commonly used in object detection and segmentation workflows. The VOC format provides bounding box annotations and object class labels, while the R-CNN Mask format includes pixel-wise segmentation masks, ensuring compatibility with Deep Learning models for both object detection and instance segmentation.

In the next stage, we trained a Mask R-CNN model over 20 epochs, using a batch size of 8 over a ResNet-50 architecture. Mask R-CNN has demonstrated its effectiveness in similar archaeological contexts^34^, including the detection of burial clusters across the Mongolian steppe^35^, burials^36^ and charcoal hearth sites^37^ in Germany, as well as terraces in Samoa^38^. The ResNet (Residual Network) backbone is a convolutional neural network architecture based on stacked residual blocks that has become a standard in Deep Learning for image analysis^39^. The model was trained and ran using the Deep Learning Libraries in ArcGIS Pro, on a Dell Precision T7820 workstation with an Intel Xeon Gold 5218 R CPU, 96 GB RAM, and an NVIDIA Quadro P2200 (4GB) GPU. Model inference was subsequently carried out using multiple confidence thresholds (0.90 and 0.95), and performance was evaluated using standard object-detection metrics. To ensure an unbiased evaluation, the dataset was partitioned into 60% training, 20% validation, and 20% test data. These data were geographically non-overlapping, and no image tiles or annotations from the validation or test areas were used during model training. The test data were fully held out and only used for final performance assessment after model training and threshold selection, ensuring independence between training and evaluation data.

### UAV-based photogrammetry

Over the central part of the research area, systematic UAV flights were conducted at 80 m above ground level (AGL) with a DJI Mavic Air 2 and a DJI Air 2S, with a velocity of 8.3 m/s, a flight velocity of 8.3 m/s, and image acquisition set to 75% frontal and 75% side overlap. While Shuttle Radar Topography Mission (SRTM) elevation data is commonly used for terrain-following flight planning, this was not always feasible in the rugged terrain of the study area. In locations where the 30 m resolution of SRTM data was not sufficient, particularly along steep cliffs, manual flights were conducted, with photographs taken at regular intervals to ensure consistent coverage. In total, approximately 13,000 RGB images were collected. Structure from Motion (SfM) processing followed a workflow modified after Peters and Stek^22^, using Agisoft Metashape Professional 2.0.0 build 15597 (64-bit). Image alignment was performed with the High Accuracy setting, with no key point or tie point limits. Following the creation of the sparse point cloud, camera alignment was optimised to improve accuracy and minimise distortion. The point cloud variance was calculated, and points with high reprojection error, high reconstruction uncertainty, or low projection accuracy were filtered out to prevent the introduction of outliers in later processing stages. No bounding box was applied, in order to retain the largest possible area. A dense point cloud, mesh, and texture were then generated, followed by the creation of a digital elevation model (DEM) and orthomosaic. The resulting orthophotograph was imported into ArcGIS for visual inspection and comparison with satellite imagery in areas where the Mask R-CNN model identified potential features. This process enabled validation of the automated detection results and offered a more detailed and up-to-date view of the current situation on the ground, complementing the older satellite imagery and allowing assessment of any recent changes.

### Pedestrian survey

Between 2012 and 2024, combined systematic and selective pedestrian surveys were conducted^11,13^, during which archaeological features were recorded using handheld GPS devices. Survey design was explicitly guided by landscape geomorphology, and different strategies were applied according to the occupational patterns under investigation. A strong component consisted of participatory mapping with community members to define survey coverage and identify areas of archaeological interest. Through this methodology, we documented local toponyms for different places. Toponyms are place names derived from topographical features and function as societal artefacts that reflect the historical origins and uses of specific locales. For agro-pastoral occupations, the survey was initially guided by information provided by local informants regarding the presence of architectural structures, which were subsequently verified through on-site inspection. In addition, systematic walks were conducted along circulation routes and across key landforms, achieving approximately 80% coverage of the geomorphological units defined for this occupational mode. For hunter–gatherer occupations, systematic transects were implemented across selected geomorphological units, reaching an estimated coverage of 60%^11^. These surveys focused on recording surface artefact concentrations and associated structural features. Architectural elements were prioritised because they are clearly identifiable in the satellite imagery employed in this study and exhibit high visibility in the field, allowing for consistent detection and spatial validation across both remote-sensing and ground-based datasets.

Throughout the research, landowners were consulted to access private lands, reflecting the importance of community collaboration and ethical engagement towards a co-produced cultural heritage protection^14^. The surveys were conducted within a 10 km radius of the present-day town of Cusi Cusi, a distance that can be covered on foot in a day. We additionally surveyed the Paicone region (Fig. 1). We acknowledge that this spatial delimitation does not necessarily reflect the broader geographical extent of pastoral mobility systems, which may operate at much larger scales. Rather, this boundary represents a methodological and operational constraint defined by the temporal and logistical resources available for fieldwork, and potential spatial bias is therefore recognised as a limitation of the study. After each field season, a geodatabase was created using QGIS (v. 3.36.0 Maidenhead) for analysis and interpretation. For this study, we focused on sites with clear architectural features. This methodological choice was driven by the objectives of the research, which seek to characterise agro-pastoral land-use practices through features that are both detectable in satellite imagery and consistently recognisable during field survey. Although this focus introduces a bias towards more durable and repeatedly reused structures, it provides a robust and verifiable dataset for linking remote-sensing outputs with ground-based observations. Furthermore, architectural features constitute one of the most reliable proxies for pastoral infrastructure in the region, where relative chronologies are often blurred by long-term reuse. We acknowledge that this approach does not fully capture finer-scale evidence such as low-density artefact scatters or small domestic enclosures; however, prioritising such architectural elements was considered an operational compromise to maximise analytical consistency and comparability within the temporal and logistical constraints of the project. We developed a typology to classify the structures, based on the possible functionalities and its relation with the *landscape logics* mentioned above^13^, resulting in 13 types. Similarly, each type of site was associated with the different logics mentioned above.

## Results

### Machine learning detection

The trained model was run across the 1750 square kilometres area of interest over the course of a day. Using the trained Mask R-CNN Object Detection Model, the automated detection phase with a confidence threshold of 0.90 yielded 572 detections, and with a confidence threshold of 0.95 it identified 471 potential enclosures across the study area. To assess model performance and determine an appropriate operating point, inference was conducted at the two different confidence thresholds on both validation and independent test datasets. Model performance was evaluated using standard object detection metrics, including true positives, false positives, false negatives, precision, recall, and F1-score. The resulting confusion matrices summarise the trade-off between detection sensitivity and false positive rates (Table 1). Recent work on machine learning in archaeological remote sensing has shown that limited labelled training data, class imbalance and the accuracy paradox are common problems when rare archaeological targets are detected within large and complex datasets^40–42^. The low precision and F1 scores reported here should therefore be interpreted in relation to the rarity of enclosure features and the visually variable background class. The confusion matrices provide the relevant basis for threshold selection: the 0.90 threshold favours recall, whereas the 0.95 threshold reduces false positives but increases false negatives, particularly on the test dataset. Because the primary aim of this study is to indicate the broader spatial extent of anthropogenic activity across the landscape and to support pedestrian surveys, we adopted the 0.90 threshold as the main operating point. In this context, favouring recall over precision is preferable, as missed detections reduce the value of the results for survey planning and landscape interpretation. The resulting potential increase in false positives is acceptable, as these detections are treated as candidate locations for prioritised field verification rather than as definitive feature identifications.

**Table 1:** Model performance on the validation and test datasets at confidence thresholds of 0.90 and 0.95.

Dataset	Threshold	TP	FP	FN	Precision	Recall	F1
Validation	0.90	51	165	21	0.24	0.71	0.35
Validation	0.95	50	134	22	0.27	0.69	0.39
Test	0.90	56	73	17	0.43	0.77	0.55
Test	0.95	47	49	26	0.49	0.64	0.56

**Fig. 5:**
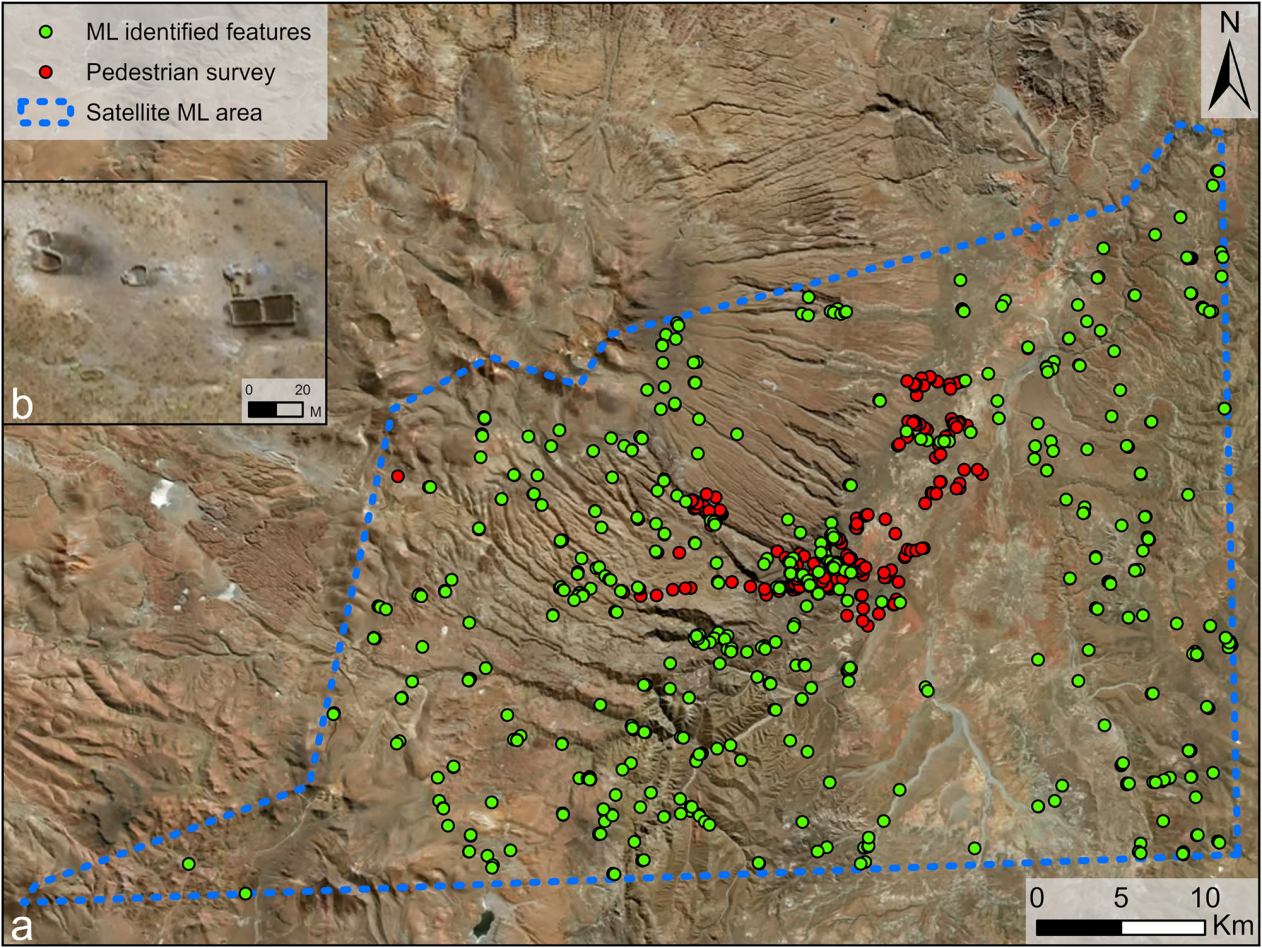
Features identified by machine learning (ML) in the research area. The Cusi Cusi research area is shown in (**a**), with green dots marking features identified through ML, red dots marking sites recorded during pedestrian surveys, and the blue dashed line marking the area covered by satellite-based ML. Inset (**b**) shows examples of different enclosure types identified in the same area, including square, round, irregular, and multipart enclosures. Background imagery: Maxar.

The ML run provided an initial set of detections, which was subsequently subjected to manual data cleaning, in which False Positives were removed, and additional True Positives were incorporated based on visual inspection and contextual cues, as well as comparison with UAV-based documentation and pedestrian survey data. Detections that were clearly inconsistent with enclosures were discarded during this interpretive step. At the selected confidence threshold (0.90), the model produced 572 candidate detections; 182 were removed as false positives during manual review, leaving 390 detections confirmed as true positives. In addition, 162 enclosure features that were missed by the model (i.e., false negatives relative to the raw detections) were identified during manual inspection and added to the interpretation layer. The majority of the False Positives were caused by naturally occurring features in the landscape that mimicked the geometry of enclosures, such as square-shaped rock outcrops and formations along riverbeds, which the algorithm mistakenly classified due to their visual similarity to anthropogenic enclosures. The final cleaned dataset consists of 552 enclosure features (Fig. 5), and represents an ML-assisted map of enclosure features across the wider study area. The detections were initially suggested by the model but subsequently reviewed and corrected through human interpretation. During visual inspection of the ML results, other types of anthropogenic features were identified for pedestrian survey, most notably 33 agricultural terraces. The final combined result constitutes a mapping product that serves as a baseline for further spatial and contextual analysis.

### UAV-based photogrammetry

**Fig. 6:**
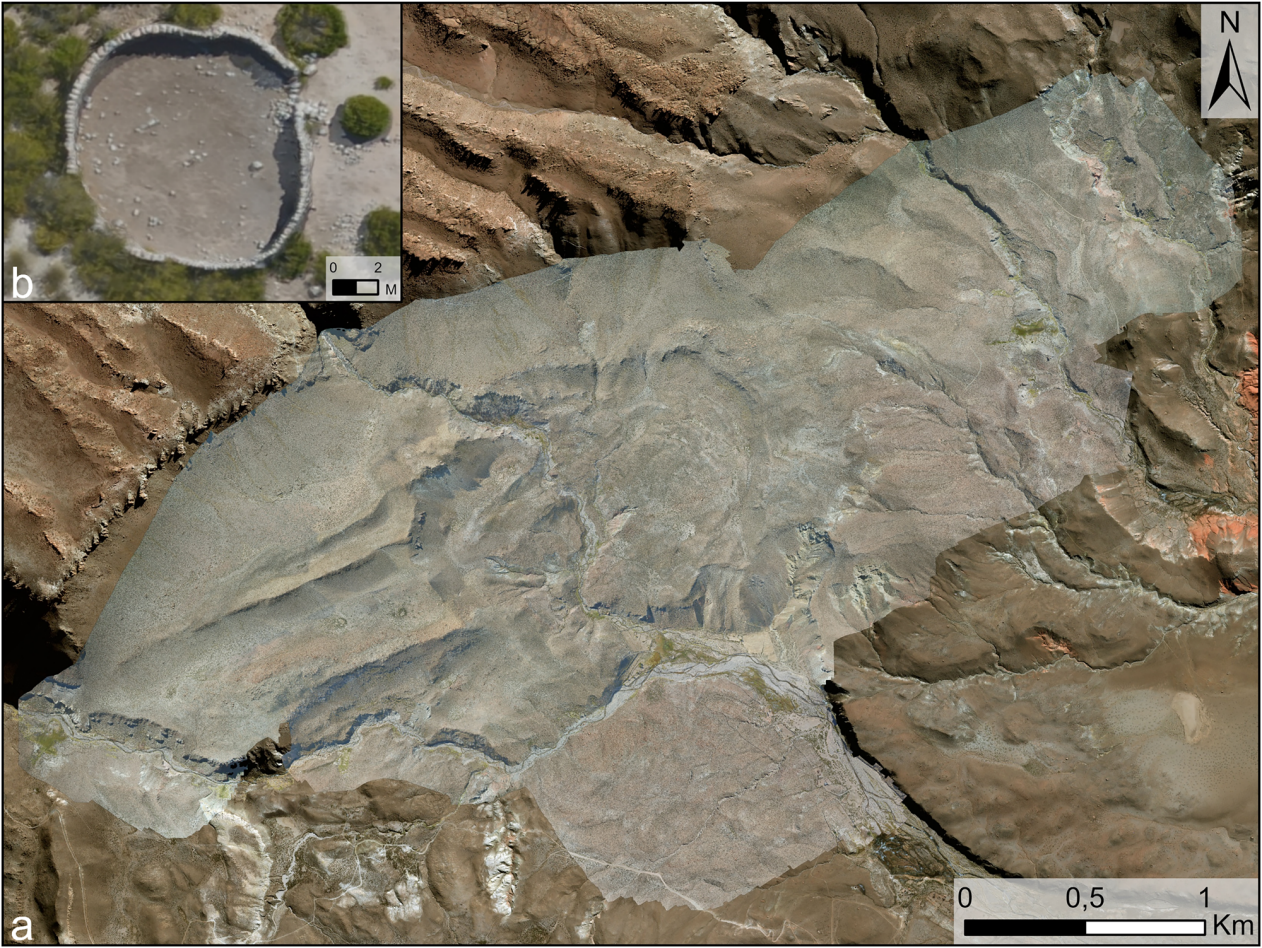
Unpiloted aerial vehicle (UAV) orthophotograph overlaid on Maxar imagery. The UAV coverage is shown in relation to the surrounding Maxar satellite imagery in (**a**). The inset in (**b**) shows a livestock enclosure visible in the UAV orthophotograph. Background imagery: Maxar.

**Fig. 7:**
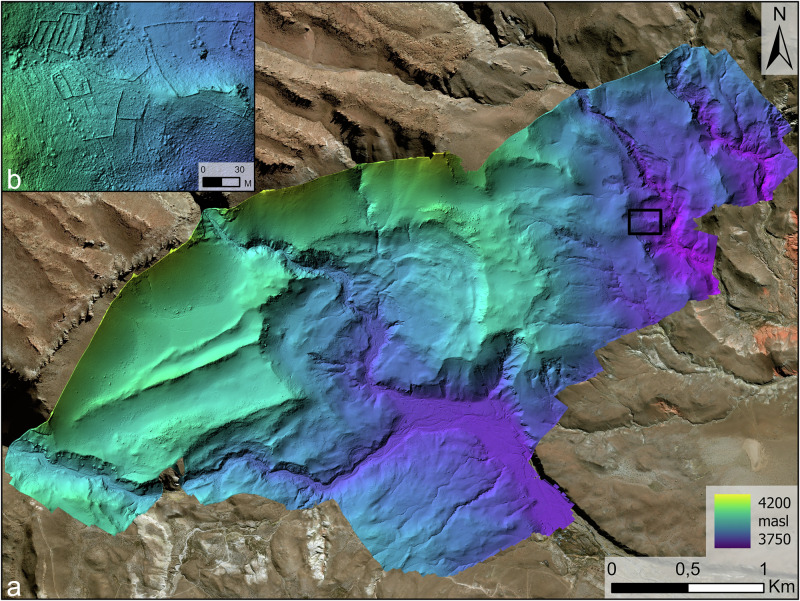
Unpiloted aerial vehicle (UAV) digital elevation model (DEM) overlaid on Maxar imagery. The UAV-derived elevation data are shown in relation to the surrounding Maxar satellite imagery in (**a**). The inset in (**b**) shows agricultural terraces visible in the DEM. Elevation ranges from 3750 masl in purple to 4200 masl in yellow. Background imagery: Maxar.

**Fig. 8:**
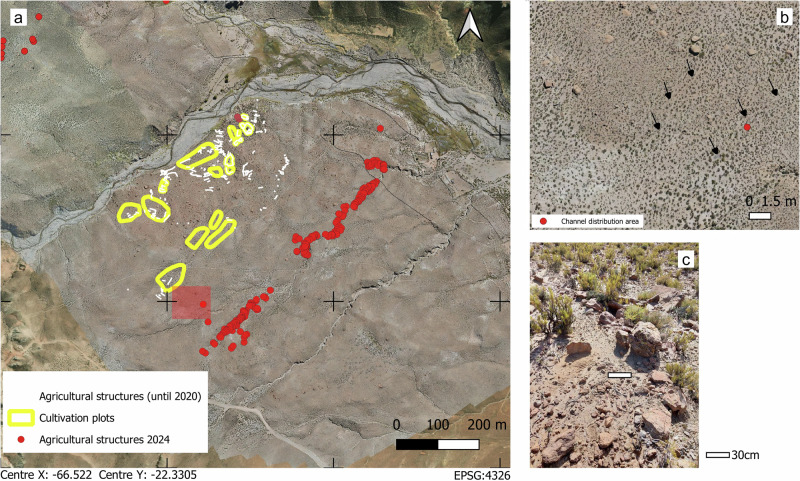
Agricultural structures, cultivation plots, and channels in the Casas Quemadas site complex. Agricultural structures recorded up to 2020 are shown in white, cultivation plots in yellow, and agricultural structures recorded in 2024 as red dots. The red rectangle in (**a**) marks the channel distribution area shown in (**b**), where black arrows mark channel segments identified through unpiloted aerial vehicle (UAV) imagery. A portion of the channel located in this area is shown in (**c**). The UAV orthophotograph is overlaid on Maxar imagery.

The UAVs were used to obtain high-resolution imagery of structures surveyed in previous years in the central region of the research area, including the wider areas of Casas Quemadas and Huayatayoc. After processing, the resulting rasters covered an area of 8 square kilometres. The orthophotograph had a resolution of 3.5 cm (Fig. 6), the DEM a resolution of 8 cm (Fig. 7). During 2023 and 2024, new agricultural structures were identified at both sites, some of which were located within the UAV imagery area. In the present study, the UAV data is used for visual analysis and comparison with existing site planimetry, as well as validating the ML data. Although our study focused specifically on detecting architectural features, the UAV orthophotograph proved efficient in helping detect and interrelate subtle surface traits which are difficult to identify even on-site, such as irrigation channels in between previously surveyed areas. At the site of Casas Quemadas, previously undetected channels were identified that connect the main irrigation canal to the southern area of the site, where a check-dam system was documented in 2024 (Fig. 8).

**Fig. 9:**
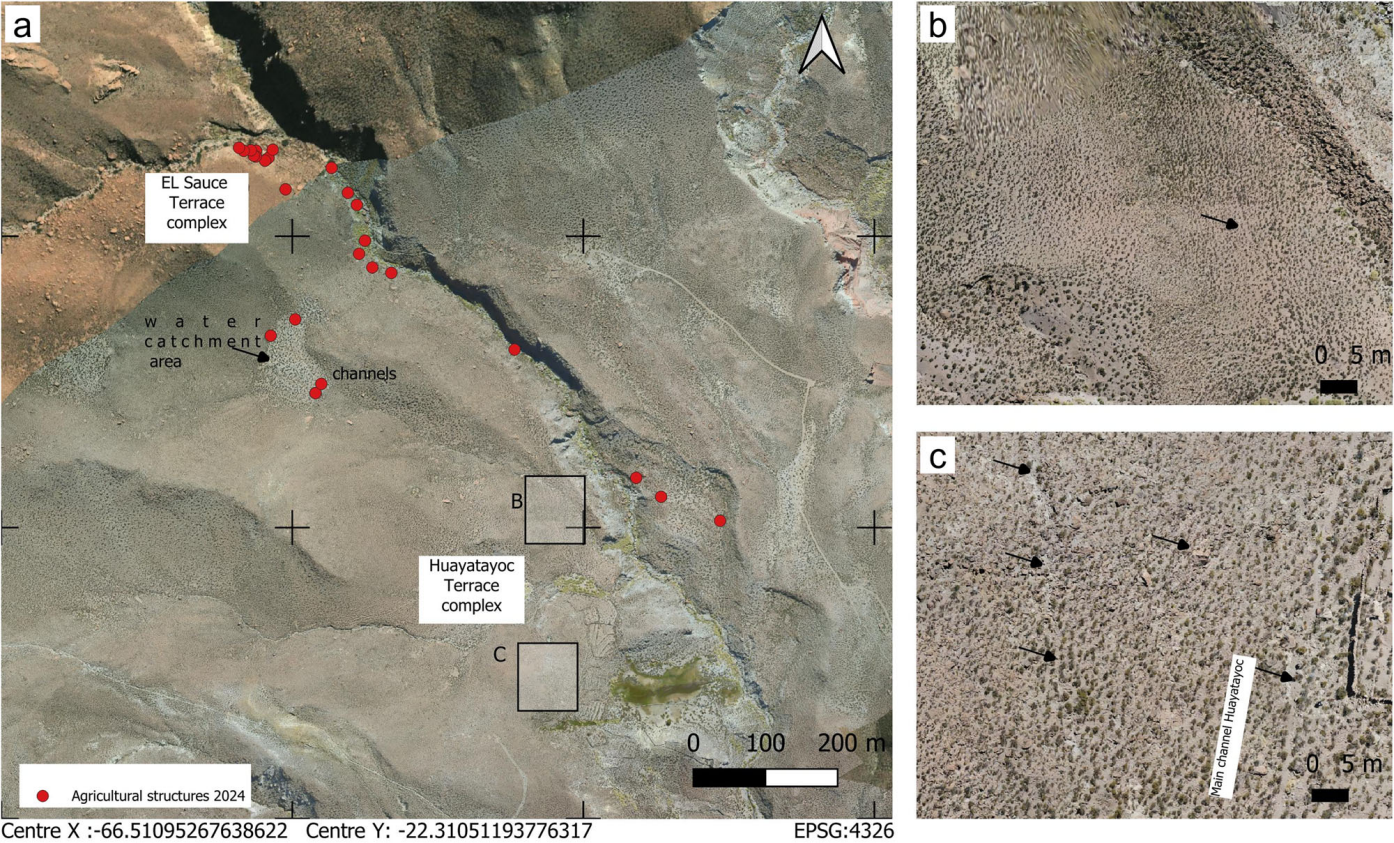
Agricultural structures, water catchment zones, and channels in the Huayatayoc and El Sauce complexes. The Huayatayoc and El Sauce complexes, their associated channels, and their connections to agricultural structures and water catchment zones detected in 2024 are shown in (**a**). Red dots mark agricultural structures detected in 2024, and black arrows mark identified channel segments in (**b**, **c**). The unpiloted aerial vehicle (UAV) orthophotograph is overlaid on Maxar imagery.

In 2024, in the region where the Huayatayoc site is located (Fig. 9), a new terrace system was detected, subsequently named El Sauce. Here, connections between the terrace system and nearby water catchment areas were identified. Pedestrian surveys in this water catchment area revealed a system of channels. Aided by the UAV imagery, it was possible to locate a channel of roughly 150 metres long, connecting the water catchment area with the southern Huayatayoc terracing system (Fig. 9, area 1). In the Huayatayoc area, it was also possible to detect additional channels that fed the main channel. In future pedestrian surveys, specific visual analyses will be performed to continue mapping the complex water management system in Casas Quemadas and Huayatayoc, to complement the existing planimetry with the available UAV imagery.

### Pedestrian survey

**Fig. 10:**
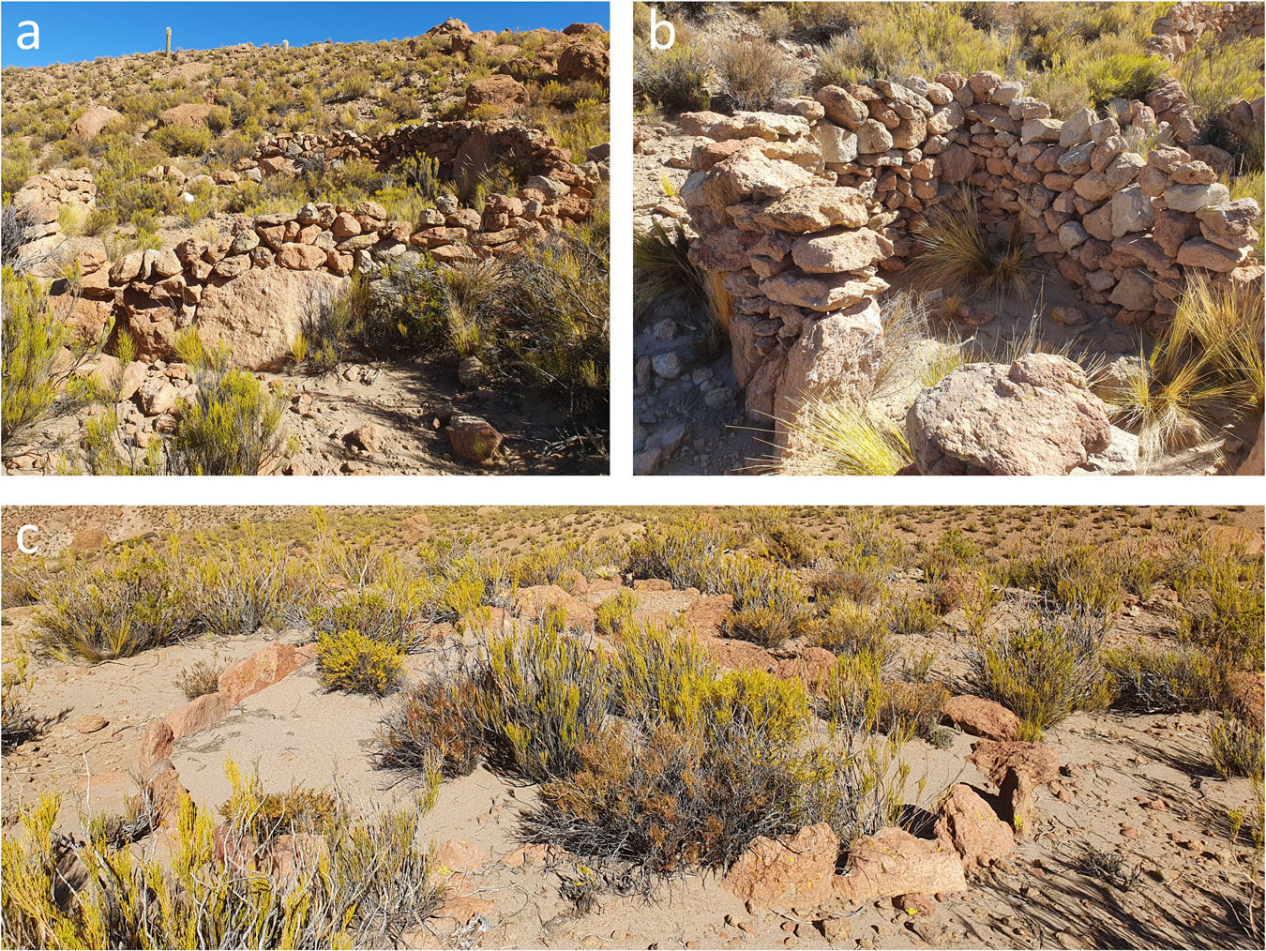
Enclosure features identified during pedestrian survey. The examples include a livestock corral (**a**), a habitation structure (**b**), and a circular structure associated with irrigation channels (**c**).

**Fig. 11:**
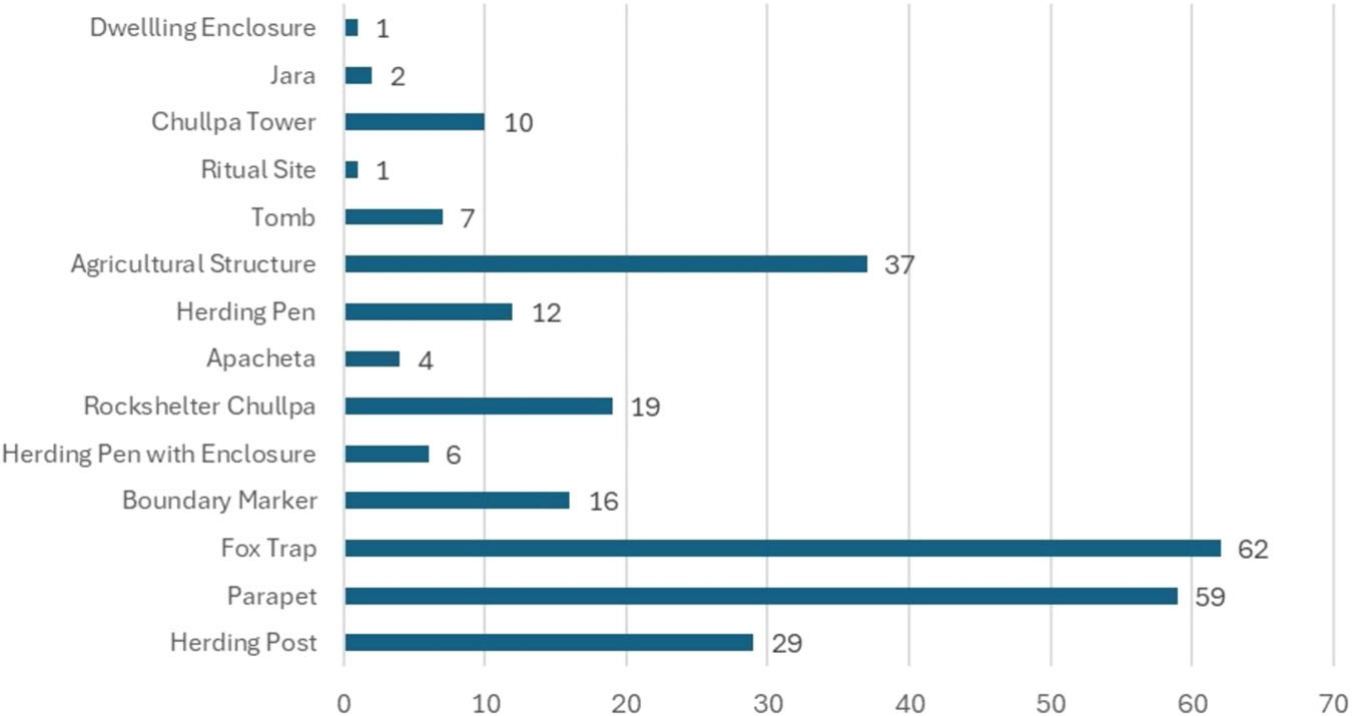
Quantity of sites per type in pedestrian surveys in Cusi Cusi and Paicone. The horizontal bar graph shows the number of sites recorded per type during pedestrian surveys in Cusi Cusi and Paicone, with blue bars representing site counts. The total number of recorded sites is 265.

**Fig. 12:**
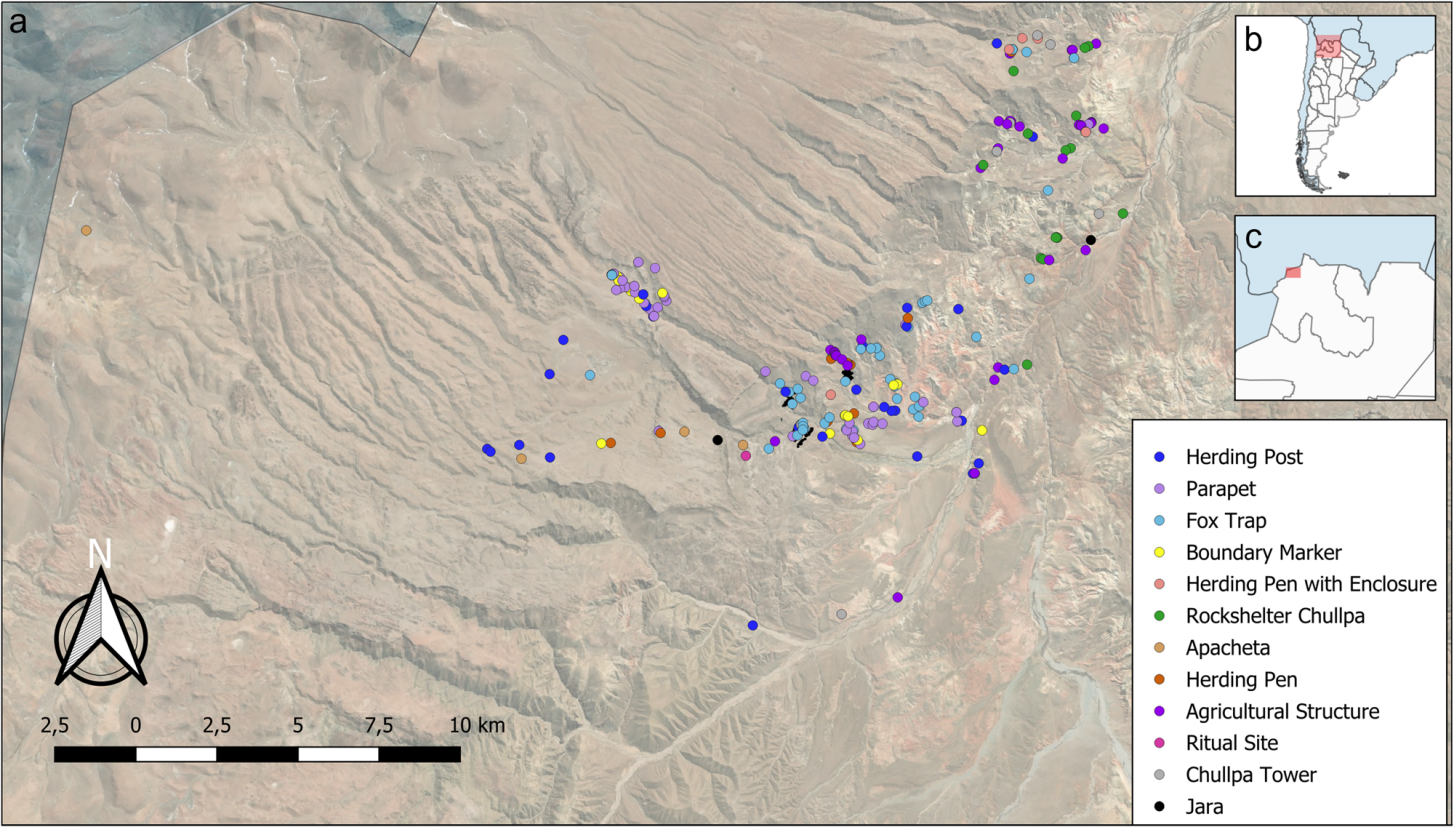
Distribution of sites per type in pedestrian surveys in Cusi Cusi and Paicone. The distribution of recorded site types is shown in (**a**), with the research area located within South America in (**b**) and within Argentina in (**c**). Site types are colour-coded as dark blue for Herding Post, violet for Parapet, light blue for Fox Trap, yellow for Boundary Marker, salmon for Herding Pen with Enclosure, green for Rockshelter Chullpa, orange for Apacheta, red for Herding Pen, purple for Agricultural Structure, pink for Ritual Site, grey for Chullpa Tower, and black for Jara. The total number of recorded sites is 265. Background imagery: Maxar.

In the pedestrian survey of the localities of Cusi Cusi and Paicone, a total of 265 archaeological sites were surveyed and documented between 2012 and 2024. These sites were recorded through direct field observation using handheld GPS units and photographic documentation (Fig. 10). The survey focused on identifying surface-visible features across different environmental settings, including valley bottoms, slopes, and high plains. These were classified based on their morphological characteristics and functional attributes (Table 2), and are distributed by type (Fig. 11). Numerous sites showed evidence of reuse and modification over time, reflecting long-term continuity in land use practices. These findings confirm the presence of a dispersed but persistent occupation across the survey area, with a strong emphasis on pastoral and agropastoral strategies (Fig. 12). The survey results constitute a crucial reference dataset for validating remotely sensed features and for interpreting broader landscape use patterns over time.

**Table 2:** Typology of structures based on previous pedestrian surveys (*N* = 188). Local Quechua language terms in italics.

Type	Category	Description	Quantity
1	Livestock outpost	Remote or smaller post used for livestock management	25
2	Parapet	Low protective wall or barrier associated to llama caravans and hunting stations	55
3	Fox trap	Stone structures used to trap foxes	52
4	Boundary marker	Stone walls that mark land ownership	16
5	Herding pen with enclosure	Herding structure with stone enclosure	2
6	Rock shelter *chullpa*	Funerary mud and stone structure located in rock shelters	4
7	*Apacheta*	Mound of stacked stones in the Andes, traditionally built as a ritual offering, often serving as a sacred landmark for travellers	4
8	Herding pen	Structure to gather livestock	9
9	Ritual site	Ceremonial site (i.e. associated with burials, offerings, monoliths)	1
10	Agricultural structure	Agricultural terraces, cultivation plots	8
11	*Chullpa* tower	Stone-walled funerary structure (empty or containing human remains)	1
12	*Jara*	Caravan station	2
13	Grouped *chullpas*	Group of funerary structures	6

### Comparison of the ML analysis with UAV and pedestrian surveys

The ML run provided an initial set of predictions, with the final total of 552 features reflecting the manually validated and corrected dataset. Since the training data contained walled enclosures of various types, the detected features included livestock enclosures, agricultural fields, and terrace walls. In the central part of the territory (Fig. 3), which was surveyed on foot, there are 19 newly identified features, of which eleven correspond to Herding Posts, one to a Herding Pen, four to Agricultural Structures, one to a Parapet, and two to Rockshelter Chullpas. Most of these have been in use for many generations. In the latter two cases, one was reused as a Herding Post with associated enclosures, while the other is part of the El Sauce site, which features a wide variety of agricultural structures.

UAV-based photogrammetry was used for visual analysis and comparison with existing site planimetry in the central part of the study area. As documented above, UAV imagery supported the identification of agricultural structures and water management features, including channels and terrace systems, particularly in areas where satellite imagery alone was insufficient for clear visual interpretation. The UAV data therefore provide complementary high-resolution visual information for understanding the spatial context of features identified through ML and pedestrian survey.

The additional pedestrian survey carried out to validate the results of the ML procedure confirmed several results of the ML and UAV work, and added a range of additional features to the ML and UAV- based dataset, including retaining slope walls and check-dams. Several ceremonial platforms, along with approximately 30 rock accumulations resulting from field clearing and previously undocumented terrace systems, were also identified. These results highlight the value of combining automated detection and UAV interpretation with pedestrian survey to build a more comprehensive understanding of the cultural landscape.

Since the pedestrian survey covered only a fraction of the wider study area, a direct quantitative comparison between the ML detections and ground results is not possible. Instead, ML detection, UAV-based photogrammetry, and pedestrian survey were applied in a complementary manner. Pedestrian survey documented and validated features within accessible areas, UAV imagery provided high-resolution visual information in selected zones, and ML detection extended enclosure identification across the wider landscape. Together, these approaches provide a more spatially extensive and detailed understanding of anthropogenic evidence in the form of enclosure distributions and associated land-use features than any single method could achieve.

## Discussion

The ML was successful in identifying a range of anthropogenic features across the wider Cusi Cusi landscape. Assigning an accurate chronological date, however, is usually complicated or even impossible with remote sensing alone, except for cases where there is a strong link between geometry and age. In the case of this study, the type of features identified by the ML workflow reflects the training dataset, as expected in supervised learning.

The training data focused on enclosure-type features, such as livestock pens and field-system walls, because these were clearly identifiable in the satellite imagery and directly relevant to the study’s aim of mapping anthropogenic activity across the landscape. Many of these structures have undergone repeated reuse over generations; however, they remain important indicators of human presence and land management. The pedestrian survey confirmed that most ML-identified features fell within this category. As one of the main objectives of this research was to document the presence and activities of Indigenous populations across the broader landscape, particularly in areas that were hard to access by pedestrian survey, the emphasis on these structures reflects a deliberate methodological choice rather than a bias. In this regard, the ML methodology showed considerable potential, as traces of human influence were visible throughout the study area, including in highly remote locations. However, this decision affects the type of evidence identified, as it concentrates on structures associated with agro-pastoral land-use systems. Other types of evidence, related to hunter–gatherer societies or even to contemporary land-use practices, are therefore underrepresented in this study. In future work, we will seek to implement strategies that allow these types of evidence to be incorporated in order to provide a more comprehensive picture of regional land-use across a broader temporal scale.

False positives are primarily associated with landscape features that exhibit geometric or textural similarities to enclosures, such as natural enclosures, erosion features, or shadow-induced patterns, while false negatives typically correspond to poorly preserved or partially visible features with low contrast relative to the surrounding terrain, including enclosures with degraded boundaries. All reported confusion matrices and performance metrics are derived from the raw model detections; no False Positives were manually removed and no True Positives were manually added prior to quantitative evaluation.

Since the study area is situated at approximately 4000 masl in rugged high-Andean terrain, large portions of this landscape are difficult or unsafe to access on foot due to steep slopes (Fig. 3), unstable rocky surfaces, long travel distances, and the presence of wildlife. As a result, a systematic pedestrian survey of the entire region is not feasible. In this context, the ML-assisted detections function as a practical tool for highlighting potential anthropogenic features, such as enclosures, that can guide targeted field verification. While not every feature can be ground-truthed, the ML-assisted results improve survey efficiency and help identify promising locations for future archaeological investigation that may clarify the chronology and long-term use of these sites.

Image chips were sampled across the full spatial extent of the study area and include a wide range of background conditions, including rocky outcrops, riverbeds, erosion features, and anthropogenic landscape elements. These landscape types are implicitly represented in the training data as background rather than as explicitly labelled negative classes, which is common practice in object-detection workflows. Nevertheless, some false positives remained in areas where natural formations exhibit enclosure-like geometry, reflecting the inherent visual ambiguity of surface features rather than the absence of representative background samples.

**Fig. 13:**
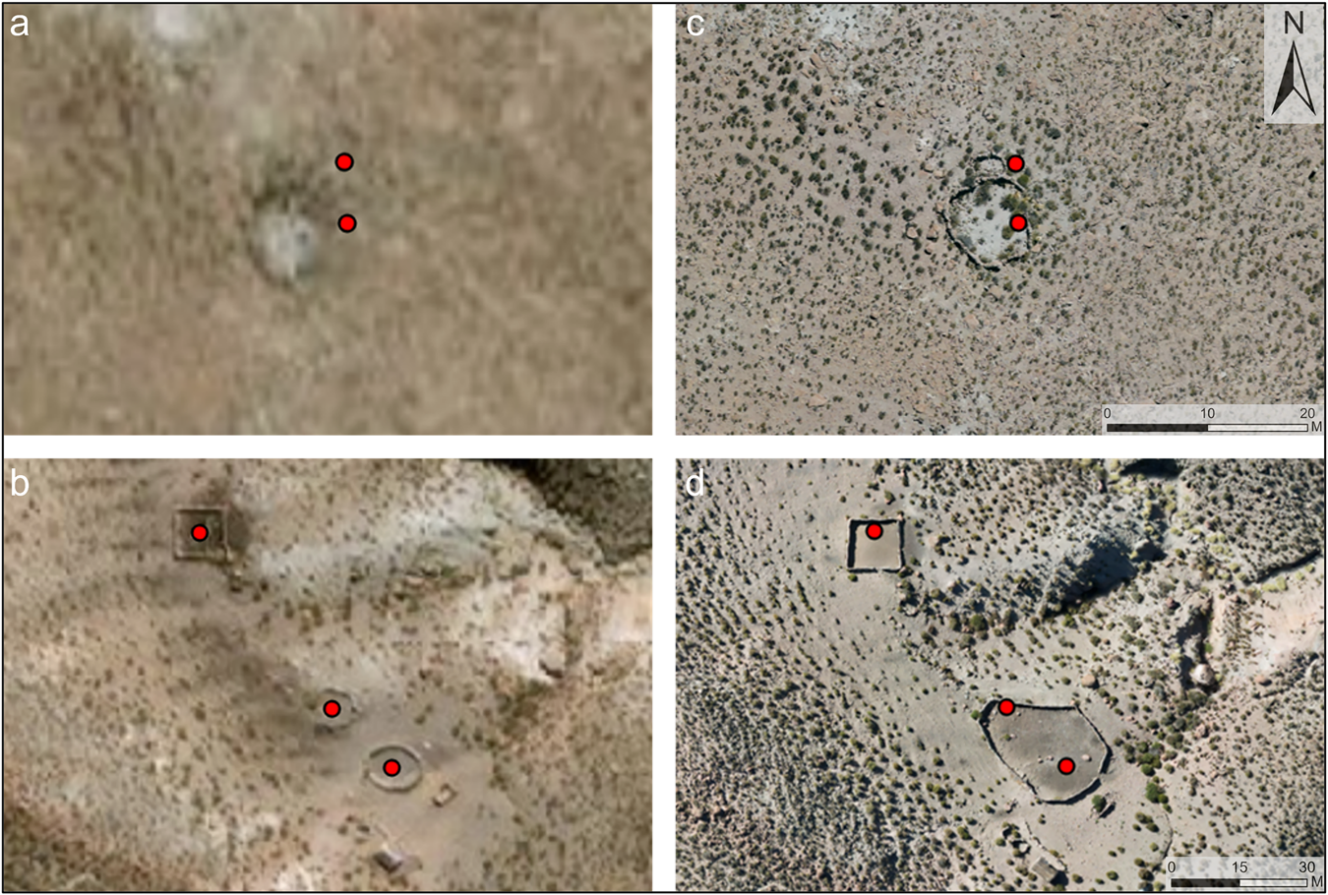
Comparison of Maxar satellite imagery and unpiloted aerial vehicle (UAV) orthophotography with feature points detected by machine learning (ML). Maxar satellite imagery is shown in (**a**, **b**), UAV orthophotographs of the same areas are shown in (**c**, **d**). Red points mark features detected through ML. The comparison highlights differences in resolution and feature visibility, while also illustrating contemporary Indigenous activities within the landscape.

The UAV work proved effective in validating and expanding the results of the machine learning analysis. In some cases, the resolution of the satellite imagery did not allow for the clear identification of features, even though the algorithm had indicated a structure at that location (Fig.13). By overlaying the UAV orthophotograph and satellite imagery, it was possible to assess the potential structures identified through the ML process, and to gain a high-resolution, current view of the layout of the sites. This also revealed that the satellite imagery was outdated in some locations. The UAV orthophotographs showed how, in recent times, some enclosures have been modified and sometimes merged into larger, singular enclosures, whereas they appeared as multiple smaller features in the satellite imagery. This is an additional way of indicating active landscape management undertaken by the local Indigenous communities across the landscape (see Fig. 13).

The large area and high altitude of the study region presented significant challenges for UAV operations. Due to field conditions and time constraints, it was often not possible to wait for optimal lighting, such as the uniform illumination provided by overcast skies during daylight hours. This limitation complicated the SfM process, which relies on consistent lighting across overlapping images for effective image matching. In addition, the high-altitude environment resulted in lower air pressure, which reduced lift capacity and made it more difficult for UAVs to maintain stable flight. As a result, it was not possible to rely solely on the onboard sensors to determine when the UAVs should return to the take-off point for battery replacement after depletion. Instead, the experience of the operators was crucial in determining safe flight durations and distances. The rugged terrain also caused unpredictable wind conditions, which could easily disrupt the flight paths of the relatively lightweight UAVs used in this study, occasionally making control difficult. Finally, the available SRTM DEM, used for the terrain following during programmed flights, has a 30 m resolution, which was not always sufficient in mountainous areas with steep slopes. Therefore, it was often essential for the operator to climb to a higher altitude and conduct the flight from there to maintain visual line-of-sight and assess the UAV’s altitude relative to the terrain. Despite these challenges, the UAV-derived results were adequate for enhancing the outputs of the satellite-based ML analysis. Although RGB photographic data proved sufficient for the purposes of this pilot study, future research could include multispectral data to identify subtle changes in land use and vegetation, and LiDAR could potentially provide a better digital terrain model in vegetated areas, although the vegetation in this region did not provide any substantial issues for this study.

The UAV imagery was not intended to provide full landscape coverage, but rather to offer targeted, high-resolution validation of selected ML-assisted detections. Flights were conducted over a central area, where the UAV-derived raster data confirmed the presence and geometry of the identified enclosure-type features, including wall alignments, enclosed spaces, and associated terrace structures, where preserved. Although not every ML-assisted detection could be validated on foot due to the physical constraints of the high-Andean environment, the correspondence observed in the surveyed zones demonstrates that the UAV results were adequate for verifying and expanding the satellite-based detections within the feasible fieldwork limits.

It should be noted that archaeological results from remote sensing are inherently shaped by the methods and parameters employed. In this study, walled features were more readily identifiable in satellite imagery and therefore formed the basis of the training dataset, whereas deteriorated terraces and retaining walls were often too subtle to detect. The pedestrian survey partially compensated for this bias by offering a complementary ground-based perspective. Some agricultural systems, particularly those incorporating terraces within enclosures, could be detected by the ML method, although these were generally previously known structures such as Huayatayoc. Looking ahead, enclosure features identified in more remote areas may represent agricultural complexes that have yet to be formally recognised. Although it depends on visual analyses, the detection of subtle features, such as irrigation channels that connect different systems, highlights the considerable potential of this combined approach. However, at present, ML alone is not sufficient for identifying such features in an automated way, due to their subtlety and poor state of preservation. Therefore, although time-consuming, the integration of ML with UAV imagery and pedestrian survey is essential for detecting these features, and significantly improves the resolution of current knowledge of water management networks.

By identifying contemporary activities across the broader landscape, this study provides clear evidence for the spatial and temporal variability of Indigenous presence and activities. Although this knowledge often exists within local communities, we now offer structured and tangible data to inform heritage management and policy. Notably, the ML method applied to satellite imagery can be extended across large areas using a similar workflow. While satellite-based data alone are not always sufficient to determine the precise nature of a feature, the combination of UAV validation and pedestrian survey has shown that when the algorithm detects evidence of human activity, it is generally reliable. Therefore, if the primary objective is to establish human presence rather than determining a detailed typology or date range, it may not always be necessary to conduct large-scale pedestrian surveys. In this way, the integrated ML approach offers a scalable method for identifying potential *loci* of activity, encompassing both past and present land use.

Our applied multiscale approach proved effective for studying large, hard-to-access areas and assessing landscape use and patterns of human occupation in this high-altitude region. Although the satellite-based machine learning procedure performed well in detecting certain features, current UAV data was essential for improving spatial resolution and enabling a more accurate assessment of present-day conditions. As with most remote sensing approaches, a targeted pedestrian ground-truthing survey was required to provide potential chronological context for the identified features. Furthermore, the results that can be obtained by ML techniques are directly dependent on the training data. In this case, we focused on enclosures as the main manifestation of anthropogenic activity in the landscape, so predominantly a large number of corrals and agricultural boundary walls were detected, reflecting the type of data used for training the ML algorithm.

As with all supervised ML approaches, the detected feature types correspond to those represented in the training dataset. In this study, enclosure-type features such as corrals, boundary walls, and associated structures were selected for training because they are the main anthropogenic expression in this research area within the high Andes, frequently reused or maintained over long periods. Their identification contributes directly to understanding long-term agropastoral activity and landscape organisation.

The inclusion of a broader range of features in the training data may result in an improvement of the model, and an increased number of identified features. In this study, the UAV orthophotographs enhanced the dataset by revealing subtle features such as degraded channels, while the pedestrian survey added further nuance by identifying retaining walls and terraces, providing a complementary perspective. The combined multiscale results suggest a long-term dispersed occupation in the Cusi Cusi region, characterised by both agricultural and pastoral activities. Beyond confirming patterns previously proposed in the literature, these findings support and add to previous studies done with different methods^10,29,31^, reinforce previously collected ethnographic data about Indigenous agropastoral practices^14^, and may contribute to Indigenous land tenure processes under Argentinian constitutional rights and territorial ancestral land rights (Argentine law No. 26.160). Moreover, this case study contributes to the growing global body of work demonstrating the value of cost- and time-effective remote sensing methods for the documentation, preservation, and management of cultural heritage^6–8^.

In the current context of the resurgence of extractivist activities in Argentina, the scale of the data presented in this study provides a valuable basis for the development of a community-based heritage database. Many of the features documented are located in areas of limited present-day circulation, making this work a significant contribution to the identification of sensitive heritage zones. In this way, the dataset offers communities an evidence-based resource to support informed decision-making processes aimed at the protection and long-term stewardship of their cultural heritage.

## Data Availability

The satellite imagery analysed in this study is publicly available. The UAV orthophotos and shapefiles generated in this study are not publicly available due to data sharing restrictions and community agreements. All relevant results are presented in the article, and further information can be obtained from the corresponding author upon reasonable request.
